# What matters for life satisfaction among the oldest-old? Evidence from China

**DOI:** 10.1371/journal.pone.0171799

**Published:** 2017-02-10

**Authors:** Sor Tho Ng, Nai Peng Tey, M. Niaz Asadullah

**Affiliations:** 1 Faculty of Economics & Administration, University of Malaya, Kuala Lumpur, Malaysia; 2 Department of Economics, University of Reading, Reading, United Kingdom; 3 Centre on Skills, Knowledge and Organisational Performance, University of Oxford, Oxford, United Kingdom; 4 IZA Institute of Labor Economics, Bonn, Germany; 5 School of Environment, Education and Development, University of Manchester, Manchester, United Kingdom; University of Glasgow, UNITED KINGDOM

## Abstract

**Objective:**

The world population is aging rapidly and the well-being of older people is of great interest. Therefore, this study investigates the determinants of life satisfaction among the oldest-old (i.e. individuals aged 80 or over) in China.

**Materials and methods:**

We use the 2011/2012 Chinese Longitudinal Healthy Longevity Survey data (*n* = 6530) for this paper. Logistic regression is used to analyse the effects of socio-demographic, economic, health, instrumental activities of daily living, family and community factors on life satisfaction and depression among the oldest-old in China.

**Results:**

Our analysis confirms the significance of many factors affecting life satisfaction among the oldest-old in China. Factors that are correlated with life satisfaction include respondent’s sex, education, place of residence, self-rated health status, cognitive ability (using mini mental state examination), regular physical examination, perceived relative economic status, access to social security provisions, commercialized insurances, living arrangements, and number of social services available in the community (*p*<0.05 for all these variables). Although life satisfaction is negatively associated with instrumental activities of daily living (β = -0.068, 95%CI = -.093—.043), and depression (β = -0.463, 95%CI = -.644—.282), the overall effect of self-rated health status is positive (*p*<0.001). This confirms the primacy of health as the determinant of well-being among the oldest-old.

**Conclusions:**

Majority of the oldest-old in China rated their life satisfaction as good or very good. Our findings show that health and economic status are by far the most significant predictors of life satisfaction. Our finding on the primacy of health and relative income as determinants of well-being among the oldest-old, and the greater influence of self-rated health status over objective health measures is consistent with the findings of many past studies. Our results suggest that efforts should be directed at enhancing family support as well as health and social service provisions in the community to improve life satisfaction of older people.

## Introduction

Asia is home to 57 percent of the estimated 900 million persons aged 60 years and older in the world, and the well-being of older people in this continent is gaining increasing attention among policy makers and researchers. There are about 210 million older adults in China, where the proportion aged 60 years and over has doubled from 7.5% in 1975 to about 15% today. The older population of China is also aging rapidly, and there are about 23 million persons aged 80 years and over.

The changing demographic structure of China has created new challenges to improve the well-being of individuals of different age groups. Market reforms in the 1990s along with the abolishment of the decades-old welfare system have created new inequalities between rural and urban residents and raised concerns about the welfare of socially vulnerable groups such as the oldest-old.

Despite decades of economic growth since 1980s, evidence indicates a significant loss in reported life satisfaction (LS) in post-reform China and only a modest recovery in recent years [1]. The burden of worsening LS has fallen primarily on the lowest socioeconomic groups [2]. Therefore, the well-being of the oldest-old, whose number is growing rapidly, deserves greater attention.

In the last three decades, China has experienced dramatic social transformations, altering earlier living arrangements. Data from population censuses show that the percentages of men and women aged 65 years and above living together with children had decreased from 67.9 and 73.6 in 1982 to 59.9 and 68.7 respectively in 2000 [3]. While the decline in co-residence with children may have alleviated conflicts and tensions, it has also reduced the familial support for the elderly, leading to depressive symptoms [4–6]. Therefore, new research re-assessing the well-being challenges for the oldest-old in China is particularly important for understanding the general role that factors such as age, sex, education, place of residence, relative income, physical and mental health, co-residence with family members and access to insurance play in shaping LS in rapidly aging societies.

Aging is usually associated with declining economic resources, decreasing cognitive ability, deteriorating physical health and weakening social support [7]. These changes in life circumstances suggest that aging might be related with declining well-being among the older adults.

Aging well requires engagement with social life, while from a personal point of view, it requires illness avoidance and maintaining physical health. Increased social participation is a positive form of adjustment to old age [8]. A recent meta-analysis of existing studies confirms poor self-rated health status and the presence of chronic disease as significant risk factors for depression among the elderly [9]. At the conceptual level, one framework explaining such links is the activity theory of aging which postulates a positive relationship between activity and LS [10]. This theoretical framework posits that “engagement with life” is an important component of successful aging. Behaviour and choices that maximize activity participation alongside avoiding disease-related disability, maintaining high cognitive and physical functional capacity are the most effective adaptive strategy in old age. In this context, it is important to understand how the relationship between health and LS is mediated by other social and economic correlates of well-being of older people in China.

There is a sizable literature on the determinants of subjective well-being in China using data from various surveys [11–14]. However, the data sets used in these studies under represent the oldest-old. Research on older population in developing countries such as China has been constrained by the lack of reliable micro data on life choices and outcomes of individuals aged 80 years and above. Moreover, most of previous research on elderly well-being lumped together all people who have been defined as older (aged 60 or 65 and above) [4–6,15–19]

To fill the lacuna of data on the oldest-old, Peking University, in collaboration with Duke University, has conducted six rounds of Chinese Longitudinal Healthy Longevity Survey (CLHLS)–in 1998, 2000, 2002, 2005, 2008–2009, and 2011–2012. An additional strength of CLHLS is that it covers rural and urban China, while well-being surveys used in the earlier research on China have tended to be disproportionately urban [2].

Data from CLHLS have been used in many studies on the subjective well-being of the oldest-old in China. These studies examined self-reported LS as a function of the respondent’s age, sex, marital status, educational level, place of residence, health status, financial source, religious participation, living arrangement, support from family members, friends or neighbours and social workers, community services and medical insurance [20–27].

Using data on the oldest-old in China from the CLHLS, we hope to contribute to the literature of the well-being of older people by focussing on this fast growing group of population. In our analysis, besides the commonly used socio-demographic and economic variables, we have also included a measure of instrumental activities of daily living (IADL), family and community factors as well as health related factors. In addition, we have examined the effects of the correlates of life satisfaction on depression, a proxy of subjective well-being. Hence, this study also aims to contribute to the literature on emotional well-being and its link with subjective well-being.

## Materials and methods

### Ethics statement

This study utilizes data from the 2011–2012 Chinese Longitudinal Healthy Longevity Survey. The survey was conducted by Peking University’s Center for Healthy Aging and Family Studies and the China National Research Center on Aging, in collaboration with Duke University, with support from the U.S. National Institute on Aging. CLHLS was conducted according to the guidelines laid down in the Declaration of Helsinki, and all procedures involving human subjects/patients were approved by Biomedical Ethics Committee of Peking University (IRB00001052-13074) and National Statistics Bureau, China (NSB Doc. No.: 2011(0008)). Permission to use CLHLS data for this paper was given by Duke University.

### Sample

The data for this study are taken from the CLHLS conducted in 2011–2012. The CLHLS is a national survey of older adults in China across 22 provinces in the country, using internationally validated questionnaires. A total of 9,765 older adults were interviewed (median age 89 years, females making up 57%, and 44% of these were interviewed in earlier waves). This study focusses on the 6,530 oldest-old adults aged 80 or over. Detailed information on CLHLS pertaining to sampling design and data quality were reported by Zeng, Vaupel, Xiao, Zhang, and Liu [28], and Zeng [29].

### Measurement

LS score was computed based on the respondents’ own rating of how satisfied they are with life on a five-point scale—very good (1) to very bad (5). Combining the few respondents who had reported their LS as very bad with those who reported LS as bad reduces the dependent variable to just four categories. It is then reverse coded for multivariate analysis, from very bad or bad (1) to very good (4). Depression was measured as a binary variable (1 if the respondent had experienced depression in the last 12 months for two weeks or more; 0 otherwise).

The independent variables in this analysis include socio-demographic characteristics, economic, health related, activity participation, family and community factors. Age and education were coded in years, male and currently married respondents were given code 1, while female and not currently married respondents were given code 0. Place of residence was classified as city, town or rural area.

The perceived income relative to others in the community was used as a proxy for the economic status of the individuals. Respondents in CLHLS were asked to rate if they were better off, same or worse off economically as compared with others in the local area. An index of ownership of social security and commercialized insurances ranging from 0 to 9 was created to measure financial security in old age. This composite index comprises retirement pension, public old-age insurance, commercialized old age insurance, public free medical services, medical insurance for urban workers, collective medical insurance for urban residents, the new rural cooperative medical insurance, and commercial medical insurance. The health variables that relate to self-rated LS include self-rated health, having physical examination once every year, depression and MMSE.

An IADL index ranging from 0 to 8 was used to measure the ability of the respondents to do the followings: visit neighbours, go shopping, cook a meal, wash clothing, walk continuously for 1 km at a time, lift a weight of 5 kg, continuously crouch and stand up three times, take public transportation. This variable measures the physical limitations of the elderly.

Living arrangement and availability of social services were hypothesized to be related with self-rated LS. Respondents were classified as living with family members or not. The total number of social services available in the community was computed based on the responses to the availability of personal daily care services, home visits, psychological consulting, daily shopping, social and recreation activities, human rights consulting services, health education, and neighbouring relations in the community.

### Analysis

Our dependent variable (LS) is an ordinal variable, hence the appropriate technique for this analysis is ordinal regression model [30]. For this purpose, we used SPSS Generalized Linear Model (GENLIN) procedure for fitting an ordinal logistic regression model to estimate the effects of the selected factors on self-rated LS. We have also used binary logistic regression to examine the effects of these variables on depression.

## Results

Table 1 reports the summary statistics of the study variables. In this survey, 17% of the respondents rated their life as very good, 46% as good, about a third reported as so-so and only 6% reported as bad or very bad. The majority of respondents were of Han ethnicity, and almost 90% were born in a rural area. The average age of the respondents in this study was 92.2 years, with about 40% in their eighties, 37% in the nineties, and a little more than one-fifth aged 100 or over. On average, female was 3.5 years older than the male. Slightly more than three quarters of respondents were widowed, while about a quarter of them were married. A higher proportion of the females were widowed as compared to the males who were more likely to be currently married. Nearly 70% of respondents did not go to school, and only 7% had at least 7 years of schooling.

**Table 1:** Summary Statistics of the Study Variables, CLHLS.

Variables	Range	Mean or proportion (SD)
***Dependent variables***		
Life satisfaction	1–4	
Very good		0.17
Good		0.46
So-so		0.32
Bad or very bad		0.06
Depression	0–1	0.14
***Independent variables***		
*Personal characteristics*		
Age	80–114	92.20 (7.70)
Male	0–1	0.40
Number of schooling years	0–24	1.57 (3.00)
Married	0–1	0.23
*Economic indicators*		
Economic status compared to others	1–3	
Better off		0.18
Same		0.66
Worse off		0.16
Social security and commercialized insurances	0–9	1.20 (0.71)
*Health indicators*		
Self-rated (subjective) health status	1–5	
Very good		0.09
Good		0.35
So-so		0.38
Bad		0.16
Very bad		0.02
Physical examination once every year	0–1	0.28
Cognitive impairment—MMSE	0–25	15.05 (8.30)
IADL	0–8	3.51 (3.08)
*Social isolation*		
Living arrangement: with family members	0–1	0.78
*Geographic factors*		
Services available in the community	0–9	1.19 (1.67)
Place of residence	1–3	
City		0.17
Town		0.30
Rural		0.53
*n*	6530	

Results from ordinal logistic regression model of LS are presented in Table 2. A positive coefficient of the parameter estimate indicates that the ordered log-odds of being in a higher category would increase while the other variables in the model are held constant. We found that respondent’s sex, education, place of residence, self-rated health, MMSE, IADL, regular physical examination, perceived economic status, number of social security and commercialized insurances owned, living arrangement, and number of social services available in the community are significant determinants of LS. On the other hand, age and marital status had no effect on LS. The lack of association between LS and age and marital status may be explained by the fact that all respondents were very old (aged 80 and over), with an ingrained outlook in life and had adjusted to being widowed.

**Table 2:** Ordinal Logistic Regression Estimates of the Determinants of Self-rated Life Satisfaction.

	Model 1		Model 2	
Variables	β	SE	β	SE
Age	0.008*	0.0037	0.008	0.0044
Male	-0.281***	0.0658	-0.308***	0.0667
Married	-0.083	0.0752	-0.075	0.0760
Number of schooling years	0.033**	0.0107	0.027*	0.0108
Economic status compared with others				
Better	2.299***	0.1077	2.259***	0.1088
Same	1.322***	0.0842	1.301***	0.0849
Number of social security and commercialized insurances	0.110**	0.0420	0.094*	0.0421
Living arrangement: with family members	0.411***	0.0714	0.407***	0.0726
Services available in the community	0.102***	0.0171	0.082***	0.0177
Place of residence				
City	0.397***	0.0821	0.374***	0.0830
Town	0.142*	0.0640	0.124	0.0643
Self-rated health				
Very good	3.787***	0.1342	3.806***	0.1379
Good	1.740***	0.0868	1.775***	0.0903
So-so	0.599***	0.0817	0.606***	0.0834
MMSE			0.025***	0.0050
Regular physical examination			0.263***	0.0652
IADL			-0.053***	0.0118
Threshold				
Self-rated LS = Very bad or bad	0.201		0.378	
Self-rated LS = So-so	3.073		3.274	
Self-rated LS = Good	5.949		6.176	
Pearson Chi-Square/df ratio	1.045		1.019	
Likelihood ratio Chi-Square	2134***		2176***	

Notes

*p < .05.

**p < .01.

***p < .001.

To fully understand the effect of self-rated health status, we estimated two versions of the life satisfaction equation. Model 1 does not contain any other health indicators besides self-rated health while Model 2 contains three additional measures of health. The estimated coefficients on self-rated health status remain stable across Models 1 and 2. This suggests that self-rated health status is not a proxy for other aspects of well-being that are correlated with health.

Model 2 of Table 2 shows that women were significantly more likely than men to rate their life as good or very good (β = -0.308, 95%CI = -0.438–-0.177, *p*<0.001). Higher educational attainment was associated with better LS (β = 0.027, 95%CI = 0.006–0.048, *p* = 0.011). Contrary to the conventional wisdom that “money can’t buy you happiness”, we found perceived economic status to play an important role in determining LS in old age (*p*<0.001). Respondents who reported their economic status as better than others (β = 2.259, 95%CI = 2.048–2.471, *p*<0.001) or same as others (β = 1.301, 95%CI = 1.138–1.465, *p*<0.001) in the same community were more likely to rate their life as good or very good, as compared to those who felt that they were worse off than others. Possession of social security and commercialized insurances tends to lead to good or very good LS (β = 0.110, 95%CI = 0.012–0.175, *p* = 0.025). There was no significant difference in the probability distribution of LS between rural and town respondents, but city dwellers were more likely to report good or very good LS as compared to those from the rural areas (β = 0.374, 95%CI = 0.213–0.535, *p*<0.001).

Co-residence with family members is positively associated with LS. Respondents who live with family members were more likely to report having good or very good LS (β = 0.407, 95%CI = 0.265–0.549, *p*<0.001). The availability of social services in the community also increases the probability of being in good or very good LS (β = 0.0082, 95%CI = 0.048–0.117, *p*<0.001). All the health variables were associated with LS, with a very strong positive correlation between LS and with self-rated health (all *p*<0.001). Respondents with more cognitive impairment were more likely to rate their life as bad or very bad (β = 0.025, 95%CI = 0.016–0.035, *p*<0.001). Contrary to expectation, LS was negatively associated with IADL (β = -0.053, 95% CI = -0.076–-0.030, *p*<0.001). Those who were more independent in their daily life activities were more likely to rate their life as bad or very bad. Respondents with regular physical examination were less likely to rate their LS as bad or very bad (β = 0.263, 95%CI = 0.136–0.391, *p*<0.001).

We repeated the regression analysis presented in Table 2 using a binary indicator of depression as the dependent variable while retaining the same set of independent variables. Estimates of the logit regression models presented in Table 3 shows that self-rated health status remains as a significant predictor of depression (*p*<0.001) after adjusting for all variables in the model). The estimated effect does not change across Models 1 and 2. Married individuals and those with better relative economic status were significantly less likely to be depressed (*p*<0.01 for both variables). While IADL is negatively associated with depression, the relationship is not statistically significant. Other factors such as sex of respondent, the number of social security and commercialized insurances, living with family members, service availability in the community and MMSE also have no significant relationship with depression. As an informal additional validation test, we re-estimated the Model 2 reported in Table 2 by adding depression as a predictor variable (Table 4). The coefficient of the dummy indicator of depression is negative and significant, confirming that being depressed also explains part of the variance in LS (β = -0.463, 95%CI = -0.644–-0.282, *p*<0.001). Those who experienced depression symptom had higher probability of having bad or very bad LS, as compared to those who did not have depression.

**Table 3:** Logistic Regression Estimates of the Determinants of Depression.

	Model 1		Model 2	
Variables	β	SE	Β	SE
Age	-0.025***	0.0066	-0.029**	0.0073
Male	-0.085	0.1070	-0.092	0.1085
Married	-0.341**	0.1236	-0.318*	0.1246
Number of schooling years	-0.025	0.0183	-0.024	0.0185
Economic status compared with others				
Better	-1.015***	0.1630	-1.069***	0.1656
Same	-0.805***	0.1097	-0.822***	0.1110
Social security and insurance	-0.007	0.0671	-0.024	0.0681
Living arrangement: with family members	-0.118	0.1098	-0.157	0.1120
Services available in the community	0.005	0.0278	-0.016	0.0289
Place of residence				
City	0.140	0.1300	0.143	0.1318
Town	-0.012	0.1041	-0.024	0.1046
Self-rated health				
Very good	-1.541***	0.1937	-1.486***	0.1983
Good	-1.694***	0.1276	-1.652***	0.1324
So-so	-1.020***	0.1086	-0.994***	0.1111
MMSE			0.001	0.0084
Regular physical examination			0.328**	0.1041
IADL			-0.036	0.0190
Intercept	2.368		2.871	
Pearson Chi-Square/df ratio	0.996		1.003	
Likelihood Ratio Chi-Square	373.5***		186.7***	

*p < .05.

**p < .01.

***p < .001.

**Table 4:** Ordinal Logistic Regression Estimates of the Determinants of Life Satisfaction (with Depression as a Regressor).

Variables	β	SE
Age	0.002	0.0047
Male	-0.278***	0.0712
Married	-0.127	0.0802
Number of schooling years	0.024*	0.0114
Economic status compared with others		
Better	2.130***	0.1185
Same	1.228***	0.0951
Social security and insurance	0.096*	0.0447
Living with family members	0.389***	0.0775
Services available in the community	0.076***	0.0189
Place of residence		
City	0.384***	0.0882
Town	0.097	0.0689
Self-rated health		
Very good	3.655***	0.1462
Good	1.653***	0.0996
So-so	0.431***	0.0928
MMSE	0.030***	0.0057
Regular physical examination	0.273***	0.0700
IADL	-0.068***	0.0126
Depression	-0.463***	0.0923
Threshold		
Self-rated LS = Very bad or bad	-0.516	
Self-rated LS = So-so	2.528	
Self-rated LS = Good	5.446	
Pearson Chi-Square/df ratio	1.007		
Likelihood Ratio Chi-Square	1892.8***		

*p < .05.

**p < .01.

***p < .001.

## Discussion

Which self-reported indicator is an appropriate measure of subjective well-being has been subject to much debate in the literature. In recent years, there is a consensus among researchers on the need to recognize that there are competing measures of well-being and that measures of LS differ from measures of more transitory “mood” [31]. Some researchers contend that Likert type multi-item scales are superior to single item scale in terms of predictive validity and reliability [32]. However, a number of studies have found that multi-items scale do not necessary outperform single-item scales under certain circumstances [33,34]. Studies comparing different survey measures find that LS score significantly out-performs other objective and subjective measures of well-being such as happiness and satisfaction with income [35–38]. Furthermore, Diener, Inglehart and Tay conclude that LS scores are influenced both by personal factors in people’s lives such as their marriage and work, as well as by community and societal circumstances [39].

Self-rated LS is a suitable measure of subjective well-being for this analysis, as we are interested to study personal as well as community-specific correlates of well-being among the oldest-old. However, we recognize that LS and emotional states are distinct subjective well-being components [18,40], and evaluative and experienced well-being are shaped differently by demographic factors such as age, gender and marital status [41]. Therefore, in addition to LS, we also assess experienced well-being in terms of depression. We include MMSE because mental health is the biggest single influence on LS and is probably more important than current income as a determinant [42]. In the developed countries, mental health problems account for over one third of disability and they can also cause physical illness. IADL is included as an indicator of physical disability [43–45].

As pointed out earlier, international research on LS has focused on health [41,46], economic factors [47,48], living arrangements [3,49] and demographic factors [50]. Studies on subjective well-being in developing countries have found relative income to be more important than absolute income [1,51–53]. Our choice of correlates of well-being in the multivariable model is guided by the aforementioned studies on China and other countries. While our models include socio-demographic, economic, IADL, family and community factors, our main explanatory variables of interest are health related factors. Following the findings of a few past studies (4,5,19,28,40), we have also examined the effects of these correlates on depression (a proxy of experienced well-being), using the same set of variables on the determinants of LS. Our approach to analysing the CLHLS 2011–2012 data is similar to that of studies on the elderly conducted in the developed countries, such as the English Longitudinal Study of Ageing by Wikman et al. [54], Piazza, Charles and Almeida [55], and Schwartz and Sprangers [56].

Consistent with the finding of Li et al. [57], we found that majority of the oldest-old in China rated their LS as good or very good, regardless of age (Tables 2 and 4), and this corroborates with findings by Wang et al. [22] and Li et al. [57], who also found no significant differences in LS between 80–89, 90–99 and 100+ cohorts in China. However, we found a distinct age effect on depression (Table 3). This contradictory finding may be explained by the fact that the oldest-old suffer from significantly higher emotional pain even though they enjoy higher evaluative well-being in old age.

Older women were much more likely than older men to be satisfied with life, and this finding corroborates that of Liu [58] and Zhou et al. [59], but contradicts that of Fong and Yen [60], and Li and Liang [61], whose studies were targeted at the “young” old in a county. This is somewhat puzzling because elderly women in China suffer from poorer health compared to men, and the gap in health between males and females increases with age despite women outliving men [62]. The result may be explained by the fact that women enjoy an advantage in adapting to old age complications than men [63].

As with the findings of Li et al. [57], we found that the oldest-old in the cities were much more likely than their rural counterparts to rate their life as good or very good. The modern facilities, good infrastructure and higher pension allowance in the cities have probably contributed to LS of the oldest-old in the cities.

Marital status was found to be an insignificant predictor of LS in this study. This finding is inconsistent with those of other studies, which found married persons to be more satisfied with their life [58,61,64]. The “anomaly” of our findings may be attributed to differences in the age of respondents, as our sample comprised the oldest-old and nearly 80% were not currently married.

The more educated oldest-old were much more likely than their lesser educated counterparts to have better LS. This corroborates the finding of Li et al. [57], although some studies have found the reverse to be true [61,65].

The negative and significant association between IADL and LS seems contradictory to activity theory. Earlier evidence using the CLHLS data shows that socially isolated elderly are more likely to encounter ADL decline compared to their counterparts [62]. Therefore our finding of the negative effect of IADL is unlikely to be driven by social exclusion since we already account for this channel. There are two plausible explanations for this anomaly. First, gains from activity are mediated through health which is adequately captured by self-rated health status in our model. For instance, Tomioka, Kurumatani, and Hosoi [66] find self-rated health status to be an independent predictor of IADL decline among older adults in Japan. Therefore controlling for the positive health effect, results in Tables 2 and 4 suggest a negative influence of IADL on LS. Second, leisure time physical activity enhances general health perceptions and physical functioning [67,68], but IADL measure in our analysis does not include activities within the realm of activity theory, as majority of the oldest-old have withdrawn from work and social activities.

Health plays an important role in self-rated LS. The significance of self-rated health status prevails even after accounting for the effects of other economic and social correlates and mental health conditions. The primacy of health as a determinant of well-being among the oldest-old and the greater influence of perceived health status over objective health measures is consistent with the findings in earlier studies on China [21,69] and other countries [46,54]. Our findings also support recent research confirming self-rated health status as an effective tool for identifying health and affective well-being of older individuals [9,66]. One challenge in the assessment of self-rated health status effect on self-rated well-being is that findings simply reflect the adverse effects of experienced well-being such as depression. Indeed, we find self-rated health status to be a significant predictor of depression (Models 1 and 2 of Table 3), which is in line with findings from recent meta-analysis of existing research on the significant risk factors causing depression among the elderly [9]. However, the absence of any evidence of self-rated health status-depression correlation explains the self-rated health status-LS gradient (see Table 4). Despite a slight reduction in the estimated coefficients, the protective effect of self-rated health status remains even after controlling for depression, and this confirms that self-rated health status is an important predictor of LS among the oldest-old in China.

According to relative income theory, an increase income will not make a person happy if the peer or his reference group has a higher increment in income as compared to him or her. The use of relative income is supported by findings from the literature that absolute income effect on happiness, while positive is smaller in magnitude compared to that of relative income effect [1,70]. We found perceived economic status to play an important role in determining LS in old age. This is consistent with other studies on the elderly population in China that also reported a positive impact of income on LS [26,58,71]. This probably captures the fact that much of the elderly health care in China today is privately financed. However, it must be mentioned that ownerships of social security and commercialized insurances were not common among the oldest-old, notwithstanding the fact that about two thirds have new rural cooperative medical insurance. Hence, there is a need to expand the coverage of social security and commercialised insurances. Efforts must be made to encourage people to start financial planning from young for old age financial security.

The traditional role of family in supporting the older persons continued to be an important contributor to self-rated LS. The feeling of being cared for among those who lived with family members would have a positive effect on LS. However, the direction of the association is a priori ambiguous. While co-residence ensures access to care and financial support, evidence also suggests that co-residence can have the opposite impact on well-being due to the fact that poor relations among household members can create tensions, conflicts and negative interaction patterns [72,73]. Some studies show that older adults living alone tend to be healthier than those who lived with others [74,75], perhaps because co-residence also comes with the added burden of looking after grandchildren which has been found to cause depression among elderly caregivers.

With the exodus of the young to the cities, family care and support are likely to be eroding in the future. Hence, there is a need to enhance community support or encourage migrants to bring along their parents to the cities. Aging parents have traditionally been looked after by their children, but in today’s China that is not always the case. Mainly due to the change in cultural norms of filial piety, the ever shrinking family size due to the one-child policy and decline in co-residence consequent upon rural-urban migration of the young, there is an increasing number of empty nesters (or left behind elderly) without family support in China. As alluded to above, more and more older men and women are not co-residing with their children [3]. The number of empty nesters is increasing rapidly in rural areas, where young people tend to migrate to cities leaving the elderly without family support and relying on farming for subsistence [76,77].

This study has reaffirmed the importance of having social services in a community [57,78]. The oldest-old who lived in a community and who have access to social services tended to have better LS than those who lived in a community with less social services.

### Limitations

One major challenge of conducting research on the perceived well-being of the very old is to obtain reliable information on self-rated life satisfaction, from those who may be suffering from loss of cognitive ability [7,79]. Moreover, the reliability of self-rated health, a very important factor affecting LS found in this study, may also vary by respondent’s education, age, economic status, and severity of health conditions [80,81]. To a certain extent, possible biases in self-evaluation of LS and health status are driven by observable socio-economic factors. Our analytical approach partly addresses the problem as the estimated regression models include rich set of controls for such factors. However, despite specifying a detailed multivariate model of LS, our analysis does not necessarily imply a causal effect of self-rated health status on well-being. The relation between health and LS can be bidirectional. While older adults with chronic illnesses can have both increased levels of depressed mood and low levels of hedonic well-being, the opposite is also true: well-being may also play a protective role in health maintenance [19,82].

## Conclusions and future research

In conclusion, to improve the LS of the old adults, attention should be paid to increase the availability of social services in the community and accessibility of health facilities. While the traditional filial piety is important for the well-being of older people, institutional provisions are needed to help the oldest-old cope with changing co-residency patterns and family-based informal care arrangements. As the social security and commercialized insurances are not popular in China and other parts of the world, it is imperative for governments to review the social protection system for the financial security for old age. However, the most important area of policy intervention is ensuring healthy aging and public provisions and increased budgetary allocations for elderly health care. Compared to high income countries and some low-and middle-income countries, China’s investment in health remains low [83]. The out-of-pocket payments still account for about 45–50% of total health expenditure, exacerbating the vulnerability of the elderly in rural areas and the poor elderly in urban areas [84]. China’s public health system remains ill-equipped to deal with long-standing chronic illness—only 2.6% of all cases of hypertension in older people in rural areas were controlled, compared to 35.1% in urban areas in China [85]. Lastly, our analysis of the determinants of depression lends support to the view that there are competing measures of well-being as evidenced from the fact that most of the factors affecting LS in our data do not matter for depression. This suggests that despite being correlated, LS differs from depression where the latter is primarily a measure of transitory mood. Therefore, we support Diener et al. [31] on the need for further research to clarify how LS and experienced well-being relate to each other. We find health status to be the most important predictor of well-being irrespective of whether we look at LS or depression. Our analysis underscores the need for future research on the oldest-old to separately study evaluative and experienced well-being to unpack the pathways underlying the persistent health effect.
